# Gene Set Enrichment in eQTL Data Identifies Novel Annotations and Pathway Regulators

**DOI:** 10.1371/journal.pgen.1000070

**Published:** 2008-05-09

**Authors:** Chunlei Wu, David L. Delano, Nico Mitro, Stephen V. Su, Jeff Janes, Phillip McClurg, Serge Batalov, Genevieve L. Welch, Jie Zhang, Anthony P. Orth, John R. Walker, Richard J. Glynne, Michael P. Cooke, Joseph S. Takahashi, Kazuhiro Shimomura, Akira Kohsaka, Joseph Bass, Enrique Saez, Tim Wiltshire, Andrew I. Su

**Affiliations:** 1 Genomics Institute of the Novartis Research Foundation, San Diego, California, United States of America; 2 Department of Chemical Physiology, The Scripps Research Institute, La Jolla, California, United States of America; 3 Department of Neurobiology and Physiology, Northwestern University, Evanston, Illinois, United States of America; 4 Howard Hughes Medical Institute, Northwestern University, Evanston, Illinois, United States of America; 5 Evanston Northwestern Healthcare Research Institute, Evanston, Illinois, United States of America; 6 Department of Medicine, Feinberg School of Medicine, Northwestern University, Evanston, Illinois, United States of America; The Jackson Laboratory, United States of America

## Abstract

Genome-wide gene expression profiling has been extensively used to generate biological hypotheses based on differential expression. Recently, many studies have used microarrays to measure gene expression levels across genetic mapping populations. These gene expression phenotypes have been used for genome-wide association analyses, an analysis referred to as expression QTL (eQTL) mapping. Here, eQTL analysis was performed in adipose tissue from 28 inbred strains of mice. We focused our analysis on “trans-eQTL bands”, defined as instances in which the expression patterns of many genes were all associated to a common genetic locus. Genes comprising trans-eQTL bands were screened for enrichments in functional gene sets representing known biological pathways, and genes located at associated trans-eQTL band loci were considered candidate transcriptional modulators. We demonstrate that these patterns were enriched for previously characterized relationships between known upstream transcriptional regulators and their downstream target genes. Moreover, we used this strategy to identify both novel regulators and novel members of known pathways. Finally, based on a putative regulatory relationship identified in our analysis, we identified and validated a previously uncharacterized role for cyclin H in the regulation of oxidative phosphorylation. We believe that the specific molecular hypotheses generated in this study will reveal many additional pathway members and regulators, and that the analysis approaches described herein will be broadly applicable to other eQTL data sets.

## Author Summary

Genome-wide association (GWA) analyses seek to relate variation of phenotype to underlying (and presumably causative) variation in genotype. Recently, many GWA studies have identified candidate genes underlying disease phenotypes such as diabetes, heart disease, and cancer risk. Many groups have also performed GWA using variation in gene expression levels as the input phenotype. These expression QTL (eQTL) studies have provided important clues as to the genetic basis of gene expression regulation. Here, we perform an eQTL study in mouse adipose tissue. We then developed a systematic analysis method to relate these patterns of eQTL associations to biological pathways. Based on this approach, we identified putative roles for thousands of candidate upstream regulators and candidate pathway members in relation to specific biological pathways. Statistical analysis showed that these predictions were highly enriched for true genetic modulators of these pathways. Based on these predictions, we also experimentally validated a role for one particular gene, cyclin H, in the regulation of oxidative phosphorylation. These findings illustrate a new analysis method for relating eQTL studies to biological pathways and identify cyclin H as a novel key regulator of cellular energy metabolism.

## Introduction

Traditional studies for mapping quantitative trait loci (QTL) have focused on identifying the causative genomic loci for individual disease-related phenotypes, such as body weight and glucose levels. Recently, microarray technologies have enabled the measurement of gene expression levels for thousands of genes in parallel, and these traits have also been used as phenotypes in genetic association studies. These expression QTL (“eQTL”) experiments have been conducted in a wide variety of organisms and cell types, including yeast, mouse (hematopoietic stem cells, brain and liver), rat (kidney and adipose), and human (lymphoblastoid cell lines) (for example, [1]–[8]). All eQTL studies to date have resulted in the identification of “cis-eQTLs” in which a strong association exists between the expression of a specific gene and the genotype at that gene's locus. Many previous studies have focused on characterizing these cis-eQTLs or using these data to prioritize candidate genes identified in clinical QTL screens.

In contrast to cis-eQTLs, associations between a gene's expression and a non-local genomic locus are referred to as trans-eQTLs. Several other groups have used trans-eQTLs to study the relationships between up-stream regulators and both transcriptional targets and phenotypic readouts [9]–[11]. These individual trans-eQTLs also organize into “trans-eQTL bands”, wherein the expression of multiple genes is associated with a single, common genetic locus. Trans-eQTL bands are commonly hypothesized to result from the differential expression of multiple downstream genes (“trans-band targets”) due to the presence of allelic variants in an upstream regulatory gene (“trans-band regulator”) found at the associated genetic locus. The functional role of trans-eQTL bands have been previously studied in yeast, in which AMN1 was shown to affect growth characteristics mediated by the transcriptional effect on several down-stream target genes [7]. Functional relationship between trans-eQTL band and biological pathways was also studied in limited set of human trans-eQTL bands [12].

In this study, we generate eQTL data from adipose tissue in a genetically well-characterized and diverse panel of inbred mice. We focused our analysis efforts on genome-wide characterization of trans-eQTL bands, testing the hypothesis that these eQTL patterns reflect known biological pathways. Specifically, we tested trans-band targets for enrichment in functional gene sets and their relatedness to candidate regulators found at the associated locus. These analyses revealed that trans-eQTL bands can be extensively mined for both previously-uncharacterized gene annotations and for novel regulators of known biological pathways. We experimentally validated one such predicted relationship, demonstrating a novel role for cyclin H in the regulation of oxidative phosphorylation.

## Results

We performed eQTL analysis in epididymal adipose tissue. This study was based on a population of diverse inbred mice, referred to here as the Mouse Diversity Panel (MDP). Recently, we and others have performed QTL analyses on individual clinical traits using the MDP [13]–[16]. Here, we used a custom mouse whole-genome Affymetrix GeneChip, GNF1M [17], to obtain the expression profiles from adipose tissue across 28 strains of the MDP. After filtering for detectable and differential expression, we identified 6601 differentially expressed genes.

**Figure 1:**
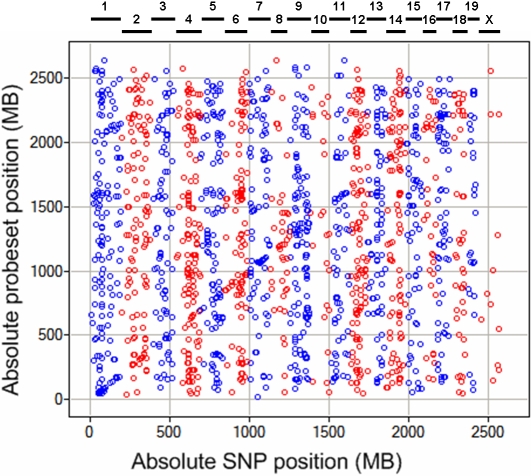
The global view of eQTL mapping results adipose tissue. The x-axis shows the absolute genomic position on the SNP/QTL axis (chromosomes shown in alternating colors), and y-axis shows the absolute genomic position of the genes whose expression was measured. Each data point in the graph represents an association between the genotype at a specific genomic locus and the expression of a gene. The strong diagonal pattern is called the “cis-eQTL band” and represents an association between the expression level of a gene and the genotype at the gene's locus. In addition, multiple vertical bands (“trans-eQTL bands”) illustrate associations between the expression of many genes and the genotype at a single genomic locus. For clarity, only data points with association scores greater than 4.2 are shown (roughly 5000 data points).

We then performed a genome-wide association analysis for every differentially expressed gene profile using our HAM algorithm, which identifies genetic associations between phenotype and inferred ancestral haplotypes while accounting for the population structure present in the MDP, as previously described [13],[18],[19]. The eQTL results are shown in Figure 1. We first characterized these eQTL data in the context of cis-eQTL associations, in which a strong association was observed between the expression of a gene and the gene's locally inferred haplotype. The presence of cis-eQTLs for many gene expression phenotypes produced a cis-eQTL band, visible as the diagonal line in Figure 1. Filtering for eQTL associations occurring within a one megabase window centered around the gene location, we identified 600 cis-eQTLs in adipose. Consistent with data reported in previous eQTL studies, the enrichment of associations around the cis-eQTL diagonal was highly significant (p<0.01) [18].

Because a large number of genes and SNPs were involved in our eQTL analysis, we first defined criteria to distinguish statistically significant trans-bands from those formed by chance. Based on permutation analysis, we conservatively required a trans-eQTL band to consist of a minimum of 50 trans-band targets for further study, resulting in the identification of 1659 trans-eQTL bands (Figure S1).

Although the assembly of individual trans-eQTLs into trans-eQTL bands was statistically significant, the biological relevance of these gene sets was unknown. To infer putative biological functions for these trans-bands, we performed a statistical enrichment analysis on the trans-band targets based on known and annotated functional gene sets (FGS). FGS were derived from Gene Ontology (GO) [20], the KEGG pathways database [21], and the Ingenuity Pathways Knowledge Base (ING) (Ingenuity Systems, Redwood City, CA).

For each trans-eQTL band, we performed a functional enrichment analysis over all FGS based on the hypergeometric distribution and a variable significance threshold (see Methods). An enrichment score (***S_e_***) was computed to reflect the degree to which a given FGS was overrepresented within the trans-band targets, and this process was repeated for all FGS. To account for the multiple-testing over all FGS, a null distribution of *S_e_* values was generated using 1000 permutations of trans-band association scores (Figure S2). This null distribution was used to compute adjusted p-values (adj. P) for FGS enrichment.

**Figure 2:**
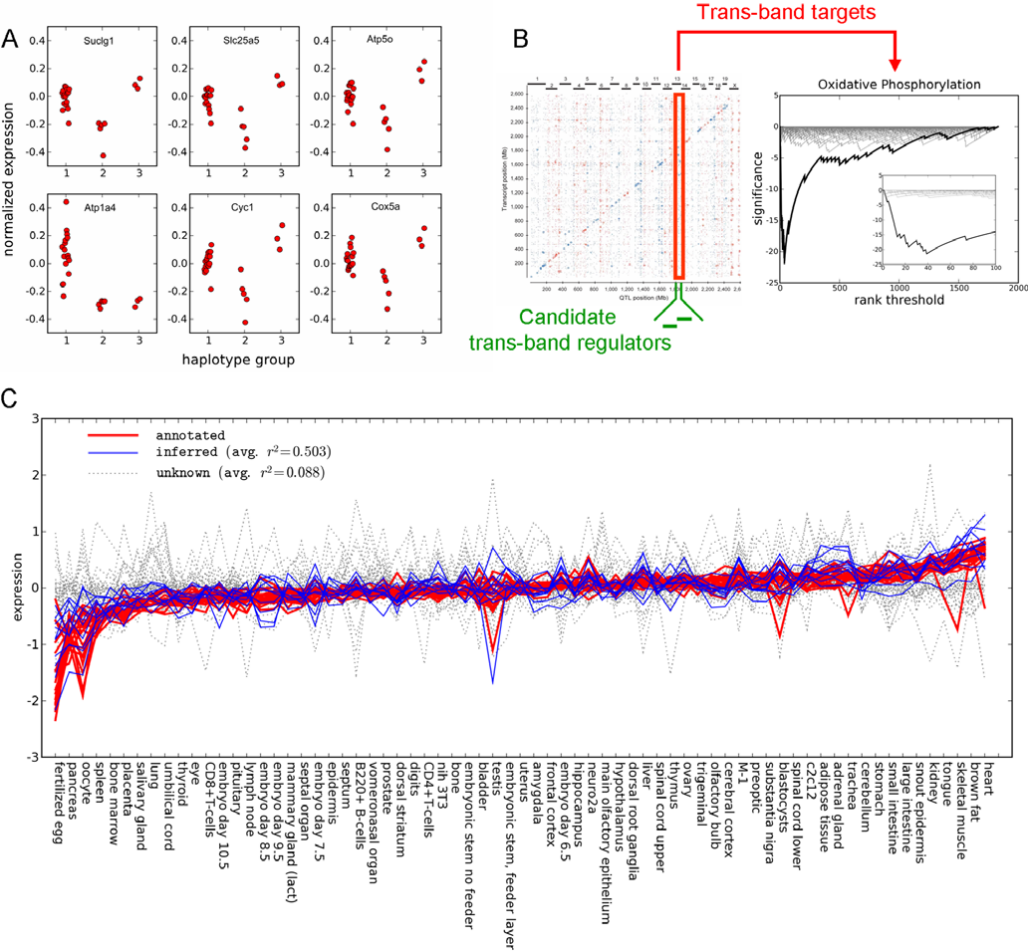
A trans-eQTL band enriched in genes in the “Oxidative Phosphorylation” pathway. (A) The expression patterns of 68 genes (“trans-band targets”) were all associated to the haplotype pattern at a region on chromosome 13 near 81.8 Mb (six strongest associations shown). Strains were assigned to haplotype groups based on local windows of genotype calls [13],[18],[19]. Each point represents the median-centered expression value on log_10_-scale for a given strain in the haplotype group. (B) Trans-band targets were tested for enrichment in FGS from GO, KEGG, and Ingenuity pathways database (ING). The genes in this trans-eQTL band were found to be strongly associated with the Oxidative Phosphorylation pathway, with the most significant enrichment (*S_e_* = −21.26) found among the top 40 annotated eQTL associations. For comparison, 100 random permutations of the eQTL association scores showed a maximum enrichment score of −4.13. Visualization of the enrichment analysis is shown as described in [39]. (C) Expression profiles in the Gene Atlas data set are strongly correlated for the 19 genes (red) which are annotated as being involved in the Oxidative Phosphorylation pathway (“eQTL/FGS genes”). In addition, 10 genes (blue, “inferred”) were also highly correlated which were not annotated in this pathway, but whose role in oxidative phosphorylation could be inferred. The remaining 39 genes (gray, “unknown”) in the trans-eQTL band do not share a correlated expression pattern in the Gene Atlas data set. Tissues are sorted from lowest to highest average expression according to 19 eQTL/FGS genes (red). All expression values have been median-centered on log_10_-scale. The “avg. r^2^” label indicates the average Pearson correlation to the genes in the eQTL/FGS group.

Among the most significant enrichments observed across all trans-bands, we identified a trans-eQTL band which was strongly associated with the category “Oxidative Phosphorylation” (KEGG, mmu00190). The trans-eQTL band was comprised of 68 genes whose expression was associated to a single locus on chromosome 13 near 81.8 MB. The six most strongly associated probe sets are shown in Figure 2A. Among these 68 trans-band targets, 40 genes were annotated in the KEGG/ING pathway databases, of which 19 genes (48%) had a previously annotated role in oxidative phosphorylation (Figure 2B and Table 1). Compared to a background occurrence of this functional annotation among genes annotated in KEGG/ING (45/1831 = 2.5%), these trans-eQTL targets were enriched 19-fold in Oxidative Phosphorylation genes, corresponding to an enrichment score *S_e_* of −21.26 (adj. P<0.001).

**Table 1:** Trans-eQTL band enriched in genes related to oxidative phosphorylation.

Probeset ID	*S_a_*	Annot a	Ox-Phos	Symbol	Description	Median GA correl.
**gnf1m03834_a**	**3.85**	**√**		**Suclg1**	**succinate-CoA ligase, GDP-forming, alpha subunit**	**0.78**
gnf1m09316_x	3.80	√		Slc25a5	solute carrier family 25 (mitochondrial carrier, adenine nucleotide translocator), member 5	0.48
gnf1m09203_s	3.65	√	√	Atp5o	ATP synthase, H+ transporting, mitochondrial F1 complex, O subunit	0.90
gnf1m32047	3.64			Atp1a4	ATPase, Na+/K+ transporting, alpha 4 polypeptide	−0.04
gnf1m04592_a	3.60	√	√	Cyc1	cytochrome c-1	0.92
gnf1m10850_a	3.50	√	√	Cox5a	cytochrome c oxidase, subunit Va	0.70
**gnf1m03550_a**	**3.49**	**√**		**Acadvl**	**acyl-Coenzyme A dehydrogenase, very long chain**	**0.69**
gnf1m05427_a	3.46	√	√	Ndufs7	NADH dehydrogenase (ubiquinone) Fe-S protein 7	0.89
gnf1m00764_a	3.42	√	√	Uqcrc1	ubiquinol-cytochrome c reductase core protein 1	0.88
gnf1m06025_a	3.41	√	√	Ndufv1	NADH dehydrogenase (ubiquinone) flavoprotein 1	0.90
gnf1m04473_a	3.38	√	√	Ndufb5	NADH dehydrogenase (ubiquinone) 1 beta subcomplex, 5	0.90
gnf1m03473_a	3.33	√	√	Atp5b	ATP synthase, H+ transporting mitochondrial F1 complex, beta subunit	0.80
gnf1m04470_a	3.31	√	√	Atp5d	ATP synthase, H+ transporting, mitochondrial F1 complex, delta subunit	0.86
gnf1m04313_a	3.29	√	√	Sdhb	succinate dehydrogenase complex, subunit B, iron sulfur (Ip)	0.91
gnf1m04837_a	3.16				RIKEN cDNA 1110008F13 gene	0.32
gnf1m04489_a	3.09	√	√	Ndufa9	NADH dehydrogenase (ubiquinone) 1 alpha subcomplex, 9	0.87
**gnf1m03340_a**	**3.03**			**Timm10**	**translocase of inner mitochondrial membrane 10 homolog (yeast)**	**0.66**
gnf1m03339_a	3.02			Timm8a	translocase of inner mitochondrial membrane 8 homolog a (yeast)	0.24
gnf1m29469_a	3.02			Cog7	component of oligomeric golgi complex 7	0.47
gnf1m00856_a	3.01	√		Bckdha	branched chain ketoacid dehydrogenase E1, alpha polypeptide	0.47
gnf1m12746_a	2.97			Arsk	arylsulfatase K	0.12
gnf1m16262_a	2.93			Coq9	coenzyme Q9 homolog (yeast)	0.59
gnf1m04426_a	2.92			Osbpl5	oxysterol binding protein-like 5	0.08
gnf1m12563_a	2.91	√		Prkacb	protein kinase, cAMP dependent, catalytic, beta	0.24
gnf1m29514_a	2.90			Clasp2	CLIP associating protein 2	0.03
gnf1m19563	2.88				Unknown	0.15
gnf1m04655_a	2.86	√	√	Uqcrfs1	ubiquinol-cytochrome c reductase, Rieske iron-sulfur polypeptide 1	0.91
gnf1m09389_s	2.85	√		Hadhb	hydroxyacyl-Coenzyme A dehydrogenase/3-ketoacyl-Coenzyme A thiolase/enoyl-Coenzyme A hydratase (trifunctional protein), beta subunit	0.55
gnf1m01006_a	2.84	√		Cyp4b1	cytochrome P450, family 4, subfamily b, polypeptide 1	0.21
gnf1m01522_a	2.82	√		Nrp1	neuropilin 1	0.01
gnf1m25672_a	2.81			Bmf	Bcl2 modifying factor	0.01
gnf1m00291_s	2.81	√	√	Cox6a1	cytochrome c oxidase, subunit VI a, polypeptide 1	0.63
gnf1m03808_a	2.79			Pex14	peroxisomal biogenesis factor 14	−0.10
gnf1m04504_s	2.79	√	√	Cox7b	cytochrome c oxidase subunit VIIb	0.85
gnf1m19612_s	2.77			AI585793	expressed sequence AI585793	0.06
gnf1m29966_a	2.76			Zfp664	zinc finger protein 664	−0.17
gnf1m32426	2.76				RIKEN cDNA 6230416A05 gene	0.27
gnf1m27063_s	2.74			Msi2h	Musashi homolog 2 (Drosophila)	0.07
gnf1m28982_a	2.74	√		Asns	asparagine synthetase	0.07
gnf1m04215_a	2.74			Gprc5b	G protein-coupled receptor, family C, group 5, member B	0.23
gnf1m00021_a	2.73	√	√	Sdhd	succinate dehydrogenase complex, subunit D, integral membrane protein	0.84
gnf1m00829_a	2.73	√		Rhoc	ras homolog gene family, member C	0.06
gnf1m28435	2.73				LOC434218, similar to Tripartite motif protein 34	−0.03
gnf1m09408_s	2.73	√		Rhoa	ras homolog gene family, member A	0.35
gnf1m29220_a	2.71	√		Fh1	fumarate hydratase 1	0.56
gnf1m19366	2.71				RIKEN cDNA A930041I02 gene	0.17
gnf1m22525	2.70				weakly similar to VESICLE ASSOCIATED PROTEIN [Rattus norvegicus]	0.08
gnf1m23009	2.70				RIKEN cDNA 2700089E24 gene	0.57
gnf1m02147_a	2.69	√		Cpt2	carnitine palmitoyltransferase 2	0.33
**gnf1m01276_a**	**2.69**	**√**		**Idh3g**	**isocitrate dehydrogenase 3 (NAD+), gamma**	**0.81**
gnf1m12534_s	2.69	√		Gja7	gap junction membrane channel protein alpha 7	−0.09
**gnf1m09960_s**	**2.68**			**Mtch2**	**mitochondrial carrier homolog 2 (C. elegans)**	**0.77**
**gnf1m03471_a**	**2.67**	**√**		**Ech1**	**enoyl coenzyme A hydratase 1, peroxisomal**	**0.63**
gnf1m30137_s	2.64	√	√	Atp5k	ATP synthase, H+ transporting, mitochondrial F1F0 complex, subunit e	0.89
gnf1m28512_a	2.64	√		Bckdhb	branched chain ketoacid dehydrogenase E1, beta polypeptide	0.44
gnf1m02816_s	2.63	√		Spr	sepiapterin reductase	0.48
**gnf1m05135_a**	**2.62**			**Mrpl12**	**mitochondrial ribosomal protein L12**	**0.72**
gnf1m07261_a	2.60	√	√	Ndufs2	NADH dehydrogenase (ubiquinone) Fe-S protein 2	0.88
gnf1m34680_x	2.60				ENSMUST00000078052 transcript (in rel.37.34e)	0.34
**gnf1m07495_a**	**2.59**	**√**		**Dlst**	**dihydrolipoamide S-succinyltransferase (E2 component of 2-oxo-glutarate complex)**	**0.65**
gnf1m11453_a	2.59			Tgoln1	trans-golgi network protein	0.33
gnf1m00838_a	2.59	√	√	Atp5a1	ATP synthase, H+ transporting, mitochondrial F1 complex, alpha subunit, isoform 1	0.83
gnf1m05094_a	2.58	√		Stx18	syntaxin 18	0.14
**gnf1m08892_a**	**2.57**			**Chchd3**	**coiled-coil-helix-coiled-coil-helix domain containing 3**	**0.71**
**gnf1m05918_a**	**2.57**	**√**		**Aco2**	**aconitase 2, mitochondrial**	**0.85**
gnf1m03972_x	2.53	√	√	Atp5j2	ATP synthase, H+ transporting, mitochondrial F0 complex, subunit f, isoform 2	0.86
gnf1m06647	2.53				cDNA sequence BC031781	−0.27
gnf1m10856_a	2.50				RIKEN cDNA 2310015N07 gene	−0.15

a Annotated in either the KEGG of Ingenuity database.

Correlation in the Gene Atlas data set was used to infer annotation for ten of the genes with no previously known role in oxidative phosphorylation (bolded).

Using this oxidative phosphorylation example as a model, we examined all trans-eQTL bands for enrichment in biological pathways. As trans-eQTL bands are hypothesized to be driven by variants of a trans-band regulator, it is expected that at least one candidate trans-band regulator should exist at each trans-band locus. In total, we identified 367 trans-eQTL bands which showed significant enrichment in at least one FGS (adj. P≤0.05) and where at least one gene was found in the associated trans-band locus (which had a median size of 44.6 kb). For example, we identified a region on chromosome 4 which was strongly associated to a trans-eQTL band enriched in genes in the tricarboxylic acid cycle, and another locus on chromosome 9 associated to genes involved in cell adhesion. In total, these 367 trans-eQTL bands were associated to loci containing 621 unique candidate genes, represented 1593 enriched pairs of trans-eQTL bands and FGS, and were enriched in 294 unique FGS (Table S1). Based on a permutation analysis over the entire set of 1659 trans-eQTL bands, we estimated that 1593 enriched pairs had a false discovery rate (FDR) of 10.5%.

**Figure 3:**
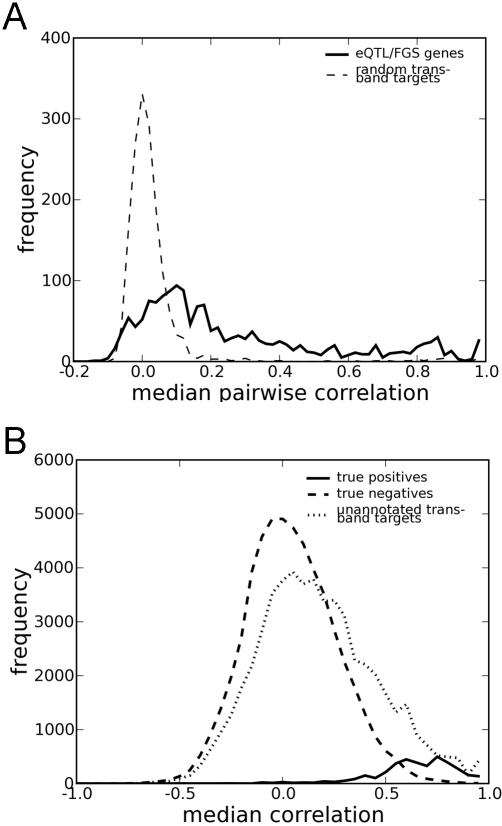
Use of the Gene Atlas reference data set to identify novel pathway members. (A) Median pairwise correlation coefficients among trans-band targets in the Gene Atlas data set were calculated for all 1593 enriched pairs of trans-eQTL bands and functional gene sets (FGS). Trans-band target genes which were annotated in the enriched category (eQTL/FGS genes) were much more likely to share a correlated expression pattern in the Gene Atlas data set than randomly chosen genes from trans-band targets. The 440 trans-eQTL bands in which eQTL/FGS genes had median pairwise correlation greater than 0.5 were selected for further study. (B) Using eQTL/FGS genes as a set of true positives, a jackknife procedure was used to calculate the median expression of each eQTL/FGS gene to the remaining eQTL/FGS genes (solid line). For comparison, an analogous calculation was performed for a presumed set of true negatives (a random set of genes) of the same size as the non-FGS genes in the trans-eQTL band (dashed line). The ratio between these two distributions was used to define a median correlation threshold of R>0.59 at 20% false discovery rate (FDR). When applied to the set of all unannotated trans-band targets (dotted line), annotation for 350 genes could be inferred.

Although these identified trans-eQTL bands were statistically enriched in genes annotated with a specific functional category, this enrichment was usually based on a small subset of trans-band target genes (an average of ∼10%) which actually shared the annotation (hereafter referred to as “eQTL/FGS genes”). Whether the other trans-band targets were not annotated with the enriched category because of spurious association, biological relationship to a distinct FGS, or incomplete gene annotation was unknown. To differentiate among these three scenarios (and to infer missing annotation in the last case), we next examined the expression of trans-band targets in a second independent gene expression data set called the Gene Atlas [17]. Whereas eQTL analysis was based on expression data from a single tissue across many strains of mice, the Gene Atlas data set measured expression of a single strain across many diverse anatomic tissues. This data set was conceptually orthogonal to the eQTL data, and we hypothesized that the Gene Atlas would be helpful in the identification of genes whose annotation could be inferred. For all significant trans-eQTL bands with FGS enrichment, we computed the median pairwise correlation coefficient in the Gene Atlas for eQTL/FGS genes (Figure 3A). For comparison, we also computed the median correlation in a random selection of trans-band targets. eQTL/FGS genes had a significantly higher pairwise correlation than random trans-band targets. Based on these data, a conservative correlation threshold of |R|>0.5 was defined for the identification of putative pathway gene sets from eQTL analysis which were also corroborated by correlation in the Gene Atlas data set. In total, we identified 440 of the original 1593 trans-eQTL band and FGS pairs which passed this threshold.

We next examined the Gene Atlas expression pattern of individual trans-band targets in this filtered set of trans-eQTL bands. To create a set of true positive observations, we performed a jackknife procedure in which each eQTL/FGS gene was successively blinded of its functional annotation. The median correlation of the jackknifed gene to the remaining eQTL/FGS genes was calculated, and this jackknife procedure was repeated for all eQTL/FGS genes (Figure 3B). For comparison, the median correlation coefficient (relative to the set of eQTL/FGS genes) was similarly computed for an identically-sized set of randomly chosen genes (a surrogate true-negative set). Based on these two distributions, we defined a threshold of median |R|>0.59 which corresponded to a FDR of 20%. Applying this threshold to the filtered set of 440 trans-eQTL bands, we inferred 2860 novel gene annotations for trans-band targets. The full set of inferred gene annotations can be found in Table S2. These inferred annotations are found in 115 unique categories and over 350 unique genes, 46 of which were not previously annotated with any functional annotation. The stringency of annotation inference could be tightened by increasing the median correlation threshold, reducing the total number of inferred annotations while decreasing the false discovery rate (Figure S3).

Following the procedure outlined above, we then reexamined the trans-eQTL band example described previously that was enriched in genes involved in oxidative phosphorylation. Like many trans-eQTL bands, we found that the 19 genes already annotated with the “oxidative phosphorylation” keyword shared a high degree of pairwise correlation in the Gene Atlas data set (median |R| = 0.86) (Table 1, Figure 2C). Consistent with their role in metabolic function, these genes were most highly expressed in heart, brown adipose, and skeletal muscle in the Gene Atlas. Of the 49 unannotated genes, 10 genes shared this expression patterns with a median correlation greater than the previously-defined threshold of 0.59, suggesting that their role in oxidative phosphorylation could be inferred from these data at 20% FDR (Figure 2C). (In contrast, the remaining 39 trans-band targets did not share correlated expression in the Gene Atlas data set, and their functional relationship to oxidative phosphorylation remained unknown.) In several cases, functional annotation inferred using this procedure was not surprising and likely represented known biology which had not yet been captured in systematic annotation efforts. For example, *Aco2* (aconitase 2, mitochondrial) and *Idh3g* (isocitrate dehydrogenase 3 (NAD+), gamma) are both involved in the generation of substrates for oxidative phosphorylation. However, in other cases these data provided the first evidence linking the function of genes to oxidative phosphorylation. For example, *Timm10* (translocase of inner mitochondrial membrane 10 homolog (yeast)), *Mtch2* (mitochondrial carrier homolog 2 (C. elegans)), and *Chchd3* (coiled-coil-helix-coiled-coil-helix domain containing 3) had no previously characterized role in oxidative phosphorylation. Coregulated expression in an adipose trans-eQTL band, combined with correlated expression in the Gene Atlas compendium, strongly implicate a previously unannotated role for these ten genes in the oxidative phosphorylation pathway.

In addition to the identification of novel pathway *members* as described above, this functional enrichment analysis was also used to identify novel candidate *regulators* of pathways enriched in specific trans-eQTL bands. In one example, we examined a trans-eQTL band which was enriched in genes belonging to the Integrin Signaling pathway as annotated in the Ingenuity database. The trans-band locus on chromosome 16 near 38.1 Mb contained four candidate trans-band regulators. However only one of these genes, *Gsk3b*, was also annotated as a member of the Integrin Signaling pathway, the enriched FGS. *Gsk3b* is a proline-directed serine-threonine kinase that was initially identified for its role phosphorylating and inactivating glycogen synthase [22]. The role of *Gsk3b* in integrin signaling is mediated through phosphorylation of its target beta-catenin and subsequent regulation of the *Wnt* pathway [23],[24], and also through interaction with *PKCdelta* as a negative regulator of *ERK1/2* (extracellular signal-regulated kinase) [25]. Moreover, *Gsk3b* is known to have a frameshift mutation near the C-terminus, which could explain the differential integrin signaling activity across the MDP. Taken together, these data strongly support the hypothesis that *GSK3b* is the regulator which is responsible for the differential expression of these trans-band targets and their putative role in integrin signaling.

In examples like these wherein the trans-band locus contained a candidate gene that shared biological annotation with the enriched FGS, that gene was considered the likely regulator of the trans-band targets. Of the 1593 pairs of trans-bands and enriched FGS, 141 were associated to a trans-band locus containing a candidate regulator matching the FGS category, as in the integrin signaling example above. Based on 1000 permutations in which each of these trans-eQTL bands was assigned to random genomic locus, the number of identified matches between a candidate gene annotation and the enriched FGS was highly statistically significant (p<0.005). Although this observation does not suggest that an upstream regulator will be found in all trans-eQTL band loci, the strong statistical significance demonstrates that this analysis enriched for true regulatory relationships which have been previously described or suggested in the literature.

Although these observations where a candidate regulator's annotation matched the enriched FGS lent confidence to the ability to rediscover known relationships, the majority of enriched trans-eQTL bands did not have such a high-likelihood candidate regulator at the trans-band locus. These cases represented potentially novel relationships between putative regulatory regions and downstream targets. Therefore, we next examined candidate regulators for the previously described trans-eQTL band enriched in genes involved in oxidative phosphorylation. The expression patterns of these trans-band targets were associated to a locus on chromosome 13 which contained two candidate regulators, cyclin H (*CCNH*) and RAS p21 protein activator (GTPase activating protein) 1 (*RASA1*). Neither of these candidate genes is itself annotated as being involved in oxidative phosphorylation. However, recent studies have implicated *MAT1*, a binding partner of *CCNH*, in the regulation of mitochondrial function and metabolic gene expression [26]. Selective deletion of *MAT1* in the heart resulted in mice with suppressed expression of genes for energy metabolism and cardiac metabolic dysfunction. Experiments in embryonic fibroblasts derived from *MAT1* conditional knockouts showed that these abnormalities were likely due to defects in *PGC1*-mediated gene expression. *PGC-1*α and β are transcriptional co-activators essential for mitochondrial energy metabolism [27]. Lack of *MAT1*, and presumably of the functional trimeric complex it forms with *CCNH* and *CDK7*, blocked *PGC-1* function. These results suggested that *CCNH* could also play a regulatory role in oxidative phosphorylation.

**Figure 4:**
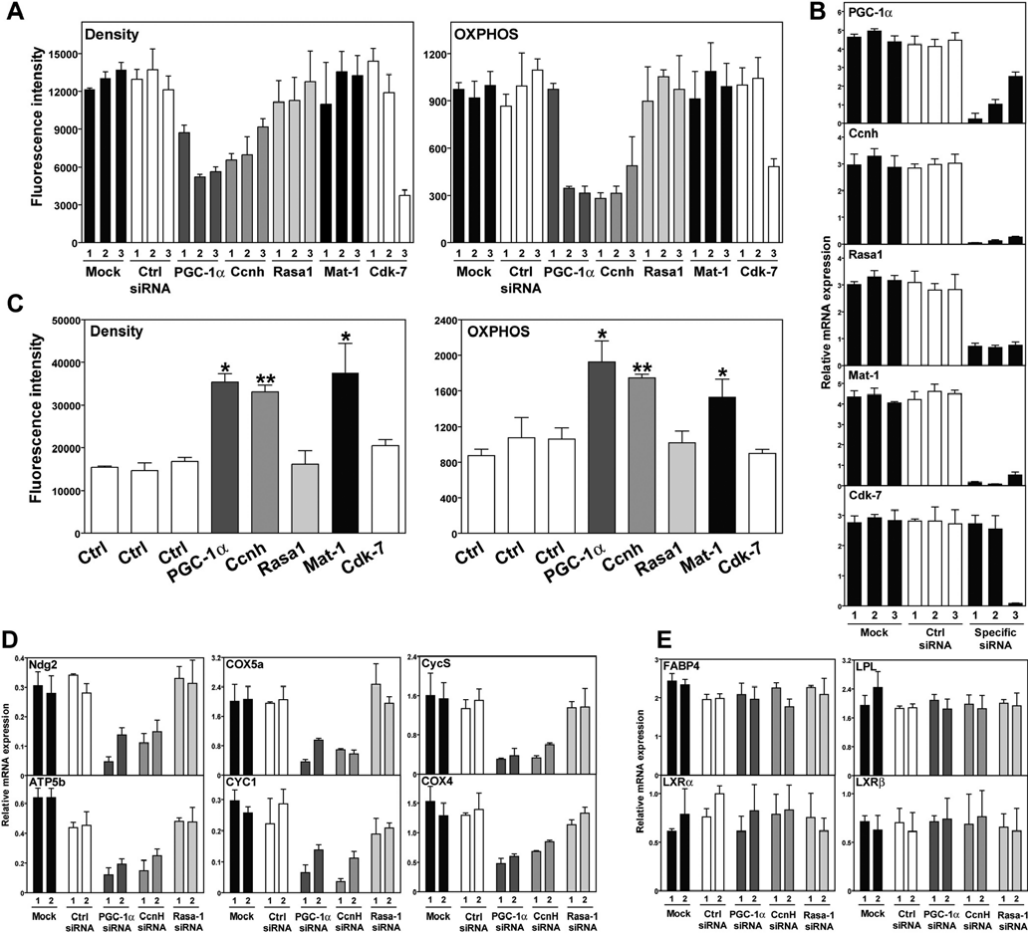
Validation of cyclin H as a regulator of oxidative phosphorylation. (A) Three individual siRNAs against two candidate genes (*CCNH* and *RASA1*) were transfected into HIB1B preadipocytes. siRNAs against *PGC-1*α and functional partners of *CCNH* were used for comparison. Mitochondrial density and oxidative phosphorylation (OXPHOS) were evaluated by FACS (see Methods). (B) RNA isolated from cells treated as in (A) was used to verify knock-down of gene expression using qRT-PCR. (C) Overexpression of *CCNH*, but not *RASA1*, resulted in increased mitochondrial activity. HIB1B cells were transfected with expression vectors for the indicated genes, and mitochondrial parameters were measured 72 hr later. (D) To confirm the effect on oxidative phosphorylation measured using Mitotracker dyes, expression of six known OXPHOS genes was assayed using qRT-PCR in response to siRNAs targeting *PGC-1*α, *CCNH*, and *RASA1*. In all cases, knock down of *CCNH*, but not *RASA1*, resulted in decreased expression. (E) To confirm that the effect of *CCNH* was not due to general effects on transcription or cell viability, the expression of four control genes unrelated to OXPHOS was also measured by qRT-PCR. In all cases, siRNAs targeting *CCNH* produced no significant change in expression.

To test the function of these two candidate regulator genes in mitochondrial function and oxidative phosphorylation, HIB1B brown preadipocytes [28] were transfected with three different siRNAs against *CCNH*. Mitochondrial density and oxidative phosphorylation were measured 72 hours later by FACS analysis of transfected cells stained with mitochondrial-specific dyes. All three siRNAs against *CCNH* induced a significant decrease in mitochondrial density and oxidative phosphorylation, similar to what is observed with knock-down of *PGC-1*α (Figure 4A). In contrast, down-regulation of *RASA1* expression showed no effect. Gene knock-down was confirmed by Taqman-based qRT-PCR (Figure 4B). *MAT1* down-regulation had no effect, in agreement with findings that this protein is dispensable for basal expression of *PGC-1* target genes [26]. In a complementary approach, *CCNH* and *RASA1* were ectopically expressed in HIB1B cells, and mitochondrial density and oxidative phosphorylation were measured by FACS 72 hours later (Figure 4C). Overexpression of *CCNH*, but not *RASA1*, resulted in significantly increased levels of mitochondrial density and oxidative phosphorylation, similar to those observed with overexpression of *PGC-1*α or *MAT1*. Overexpression of *CDK7*, the third member of the trimeric complex (*CDK7*/*CCNH*/*MAT1*) did not result in increased mitochondrial density or oxidative phosphorylation, but knock-down appeared to affect these phenotypes.

To confirm these observations in a secondary assay, we also demonstrated decreased expression of six well-characterized OXPHOS genes in response to *CCNH* but not *RASA1* (Figure 4D). It is worth noting that the magnitude of the changes observed with *CCNH* knockdown matches those observed with knockdown of *PGC-1*α, the best characterized regulator of these mitochondrial parameters. To exclude the possibility that these observations are due to general effects on transcription or cell viability, we measured the effect of *CCNH* RNAi on four control genes which are unrelated to OXPHOS but highly expressed in HIB1B cells. The knock-down of *CCNH* had no effect on their expression levels (Figure 4E).

We then sequenced through the CCNH gene in 20 of the MPD strains profiled. We discovered nine SNPs, all of which exactly matched the haplotype structure inferred from the genome-wide SNP data (Table S3). One was found in the upstream regulatory region, six were found in intronic regions, and two were found in the 3′ untranslated region. While none of these SNPs were found to produce amino acid changes in the protein product, a possible functional role of these noncoding SNPs could be mediated through the regulation of CCNH gene expression. This hypothesis is corroborated by an observed association between expression of CCNH and haplotype structure at the trans-eQTL band locus (p = 0.049), albeit at a lower significance than was used to filter the trans-eQTL band itself.

In summary, these results demonstrate that *CCNH*, not *RASA1*, plays a role in the regulation of oxidative phosphorylation and validate the use of trans-eQTL functional analysis to identify candidate regulators of pathways. Moreover, they also highlight the significance of the trimeric *CDK7*/*CCNH*/*MAT1* complex in the regulation of mitochondrial function.

## Discussion

This novel regulatory role for cyclin H in the regulation of oxidative phosphorylation was one of the 1593 trans-eQTL band enrichments detected in the adipose eQTL analysis. We believe that the remaining enrichments (Table S1) contain many promising and testable hypotheses relating candidate genes to specific biological processes. Moreover, we suggest that this approach will be generally useful for the analysis other eQTL data sets which are becoming increasingly available.

In this study, we have chosen to use a diverse inbred mouse population for eQTL analysis. This population enables utilization of and contribution to community resources for genotype and phenotype data. However, several caveats for this approach have been previously discussed [13],[18],[19]. Most notably, population structure in the MDP potentially leads to false positive associations if left uncorrected. In this study, we utilize a previously described approach for accounting for population structure that is based on a weighted bootstrap procedure [18]. Moreover, we suggest that the statistical significance of the eQTL patterns described above is robust to any individual false positive eQTLs due to residual population structure. The analysis reported here relies on patterns of eQTLs rather than individual eQTL associations, and any false positives eQTLs resulting from population structure would not be expected to assemble into trans-eQTL bands. Nevertheless, we have also performed all analyses after removing the most distantly related strains (including *CAST*/*EiJ*, *CZECHII*/*EiJ*, *JF1*/*Ms*, *MOLF*/*EiJ*, *MSM*/*Ms*, and *SPRET*/*EiJ*), thereby reducing the extent of the background population structure. In these analyses, we also found the same statistically significant results for all global permutation analyses reported above.

Although this eQTL analysis approach results in associations between the genotype of an upstream regulator and the expression of downstream transcriptional targets, it should be emphasized that many mechanisms are consistent with these results. In the simplest scenario, the upstream gene could be a direct transcriptional regulator, and all trans-band targets could be direct transcriptional targets. However, the upstream gene could also be an enzyme which activates an intermediate transcriptional regulator, which in turn modulates other trans-band targets. Or the upstream gene could have only one direct transcriptional target, which in turn results in a complex transcriptional cascade that ultimately results in a trans-eQTL band. We suspect that these and other potential mechanisms are all likely to underlie the reported trans-eQTL bands. Strategies to characterize the details of the mechanism are case-specific and not addressed in this study. Nevertheless, the specific molecular hypotheses presented here are equally valid regardless of these mechanistic details. In the case of either direct or indirect regulation, the eQTL analysis strategy presented here provides fertile starting points for relating candidate genes underlying eQTLs to specific biological pathways.

High-throughput, genome-wide expression profiling is a powerful tool for genomic studies for discovering differentially expressed or co-expressed genes. eQTL analysis extends this methodology by measuring quantitative variation of gene expression across a well-defined genetic mapping population. The functional enrichment analysis introduced here allows association between eQTLs and biological pathways, enabling the identification of both novel members and novel regulators of these pathways. This method may be particularly relevant and complementary to whole-genome association studies in large clinical populations [29]–[34]. As more candidate genes underlying polygenic human diseases are identified, the next challenge will be to understand how these candidate regulators alter susceptibility to disease. Our current data suggest that including genome-wide expression profiling across these mapping populations will enhance our ability to identify both diseases susceptibility genes and their underlying pathways.

## Materials and Methods

### SNP Data

Data from several large-scale genotyping efforts were combined to generate a high density genotyping data set based on the MDP population [13],[14],[35].This data set contained allele calls for approximately 157 k SNPs across 46 diverse inbred mouse strains. Approximately 89.7% of these SNPs have a minor allele frequency greater than 10%. The median inter-SNP distance was 6.02 kb across all autosomes (mean distance = 15.2 kb). The raw genotyping data contained 9% missing values.

Because the HAM algorithm is very sensitive to missing data, we applied a conservative imputation algorithm to infer more than half of those missing genotype calls based on local linkage disequilibrium. Missing values in SNP genotyping data were imputed based on known flanking genotype calls. Imputation was performed only if the flanking 3–10 allele window matched at least five other strains and all of these matched known genotype windows had an unambiguous genotype call at the position of the missing value. Validation of this imputation method was performed by sub-selecting SNP positions with no missing data and then randomly removing 9% of allele calls (approximately the true missing data rate). In these validation experiments, approximately 99% of imputed values matched the true allele call at a call rate of approximately 55%.

### Sample Preparation

Epididymal adipose tissue was dissected from a panel of 28 diverse inbred strains (*n* = 3 except as indicated below): *A*/*J*, *AKR*/*J*, *BALB*/*cByJ*, *BTBR T*+ *tf*/*J*, *BUB*/*BnJ*, *C3H*/*HeJ* (*n* = 4), *C57BL*/*6J*, *C57L*/*J*, *CAST*/*EiJ* (*n* = 2), *CBA/J* (*n* = 2), *CZECHII*/*EiJ* (*n* = 2), *DBA*/*2J*, *FVB*/*NJ*, *JF1*/*Ms*, *MOLF*/*EiJ*, *MSM*/*Ms* (*n* = 2), *NOD*/*LtJ* (*n* = 1), *NZB*/*BlNJ*, *NZW*/*LacJ*, *PERA*/*EiJ* (*n* = 1), *PL*/*J*, *RIIIS*/*J*, *SEA*/*GnJ*, *SJL*/*J*, *SM*/*J* (*n* = 2), *SPRET*/*EiJ* (*n* = 2), *SWR*/*J* (*n* = 2) *and WSB*/*EiJ*. All mice were maintained on a 12-h light/dark cycle at least 1 week before collecting tissues and individually housed with food and water available *ad libitum*. At 25 weeks of age, mice were sacrificed under isoflurane anesthesia by cervical dislocation at ZT 6 (ZT 0 defined as lights on) 2 h after food deprivation. Epididymal adipose was dissected, snap-frozen in liquid nitrogen and stored at −80°C for subsequent analysis. cRNA was prepared as previously described [17], and replicate samples were pooled by strain.

### Expression Profiling

The gene expression profile of adipose tissue sample was obtained from customized Affymetrix GeneChip, GNF1M, which contained 36,182 probe sets targeting over 27,000 unique mouse genes. Each expression measurement was summarized by gcRMA (in the bioconductor package) [36]–[38] from the quantile-normalized probe intensities of a probe set. Then the median expression value from each sample was adjusted to 100 (in real scale) by linear scaling. Genes were then filtered for detectable and differential expression by requiring maximum expression to be greater than 200 and the ratio between maximal expression and minimal expression to be greater than 3. The remaining differentially expressed genes were kept for the further eQTL analysis. Primary data has been deposited at NCBI GEO under the accession number GSE8028.

### Genome-Wide eQTL Mapping

The detailed algorithm underlying the Haplotype Association Mapping (HAM) method has been previously described [13],[18],[19]. Briefly, HAM uses ANOVA to calculate the strength of genetic associations between an input phenotype and the ancestral haplotype structure (as inferred using a local window of three adjacent SNP alleles across the genome). A weighted bootstrap method was introduced to detect association peaks conditional on the population structure in the MDP [18]. At each genetic locus, the association score (*S_a_*) was represented as the negative log10-transformed p-value. HAM analysis was performed for all differentially expressed genes in adipose across 28 strains. Expression phenotypes were in log scale. Unless otherwise noted, all analyses were performed based on a cutoff value, *S_a_*>2.5 (or *P*<0.003). The genomic mapping of all genes and SNPs was based on Mouse Genome NCBI Build 33 (mm5). Candidate genes were identified as any gene which overlapped the five-SNP window centered on the inferred haplotype locus described above.

### Trans-eQTL Band Size Significance

We generated 1000 permuted eQTL results by randomly picking associated loci for each gene from a pool of loci with at least one significant association. For each gene, the number of associated loci in the permuted results remained the same as in the true result. For each permutated eQTL result, the distribution of trans-band sizes was recorded. We observed that a trans-band formed by chance contains at most 29 genes (Figure S1). In the interest of setting a simple and conservative threshold, we only analyzed trans-eQTL bands with 50 or more trans-band targets (*P*<0.001). After conservative merging of locally redundant trans-eQTL bands (but without merging redundant FGS), we identified 1654 trans-eQTL bands.

### Biological Knowledge Represented in Gene Sets

The Gene Ontology (GO) database was downloaded from http://www.geneontology.org/ontology/gene_ontology.obo. The snapshot of April 03, 2006 was used in this analysis, which contains 21,316 GO terms in three categories for biological process (BP), molecular function (MF) and cellular component (CC). Three unknown categories, “GO∶0000004”, “GO∶0005554” and “GO∶0008372”, were removed for the analysis. The mapping from Entrez Gene IDs to GO terms was obtained from NCBI's gene2go table (April 03, 2006 snapshot from ftp://ftp.ncbi.nih.gov/gene/DATA/gene2go.gz). In addition, we utilized two databases of manually-annotated metabolic and signaling pathways. The KEGG pathway database was downloaded from ftp://ftp.genome.jp/pub/kegg/pathways/mmu/. The snapshot of April 26, 2006 was used, which contains 174 pathways for mouse. Ingenuity pathways database (ING) was obtained from Ingenuity Inc., which contains 137 pathways for mouse. All flat-file formatted databases were parsed by individual python scripts for the use in the functional analysis.

### Functional Analysis of Trans-Bands

Our functional analysis of trans-bands was adapted from a previously described method for gene set enrichment [39]. For each trans-band and FGS pair, the enrichment score *S_e_* is calculated by:
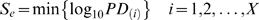
where ***X*** is the number of associated genes with *S_a_*>2.5, and
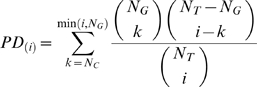
where N_G_ is defined as the number of genes assigned to the FGS, N_T_ is the total number of annotated genes in the eQTL data set, and N_C_ is defined as the number of genes out of the top *i* associations assigned to the FGS. An annotation-specific background was used for each FGS type, utilizing separate N_T_ values for BP, MF, CC, and KEGG/ING.

### Permutation for the Significance of Enrichment Scores

Our functional enrichment analysis screened thousands of FGS for each trans-eQTL band. To adjust for multiple testing, we used a permutation procedure to assess the significance of the enrichment scores (*S_e_*), which were then converted to adjusted p-values (*adj. P*):

1. For each trans-band, we generated 1,000 randomized trans-bands each containing the same number of genes as the true trans-band, sampled without replacement from the set of annotated genes in the eQTL data set.
2. The FGS enrichment analysis was applied on each randomized trans-band, and the most significant *S_e_* value across all FGS was recorded as 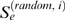, *i* = 1 … 1000. This vector serves as the null distribution of *S_e_*.
3. An *adj. P* was calculated as the percentage of random 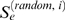 values that are more significant than the true *S_e_* value:
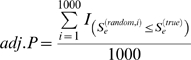
where 
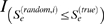
is an indicator function. The threshold for *adj. P* was set to 0.05 for the further exploration.

### Assessment of Mitochondrial Parameters

HIB1B preadipocytes were cultured in Dulbecco's modified Eagle's medium containing 10% fetal calf serum. Cells were transfected with indicated siRNAs or cDNA expression vectors (plus a GFP-expressing plasmid) using DharmaFECT 4 (Dharmacon) or Fugene 6 (Roche) respectively. 72 hr later, mitochondria were stained by adding Mitotracker Green (Molecular Probes) to culture medium and incubating cells for 30 minutes at 37°C. Similarly, oxidative phosphorylation was measured by incubation with Mitotracker CMXH_2_Ros (Molecular Probes), a mitochondrial-specific stain that needs to be oxidized in an actively respiring cell in order to fluoresce and thus provides a measure of OXPHOS. Cells were washed in PBS, trypsinized, and resuspended in Hank's buffer containing 0.5% fetal calf serum. Analysis of mitochondrial density and function was performed in 10,000 cells per sample using flow cytometry. For siRNA experiments, the entire population was included since transfection efficiency was close to 100%, as assessed using a rhodamine-labeled control siRNA. For overexpression experiments, transfected cells were identified (GFP positive population) and mitochondria mass and function evaluated exclusively in these cells. TaqMan quantitative RT-PCR was used to confirm siRNA-dependent gene knockdown. Data are representative of three different experiments performed in triplicate. Error bars indicate standard deviation. Sequences for siRNAs and qRT-PCR primer/probe sets are available upon request.

## Supporting Information

Figure S1 Histograms of the number of trans-band targets in a trans-eQTL band formed by chance in fat eQTL data. The number of trans-band targets in each trans-eQTL band was computed, and a histogram of trans-eQTL band sizes is shown in blue. For comparison, the QTL location of each association in the eQTL matrix was permuted 1000 times, and each gray line represents the histogram of trans-eQTL band sizes of a permuted eQTL result.
(0.17 MB DOC)

Figure S2 Enrichment score threshold for multiple testing correction over all FGS. At each size of trans-eQTL band (x-axis), 1000 random permutations of association scores was generated. For each permutation, enrichment calculation was performed for all FGS, and the maximum enrichment score was recorded. Recorded values were used to define enrichment score thresholds at 0.001, 0.01 and 0.05 (blue, green, and red, respectively) adjusted for multiple testing over all FGS. These background distributions were used to calculate adjusted enrichment p-values (adj. P).
(0.16 MB DOC)

Figure S3 Number of inferred annotations and false discovery rate (FDR) as a function of median correlation threshold. The median correlation cutoff was used to define a threshold of inferring missing annotation. Initial settings were chosen corresponding to a 20% FDR at 0.59 cutoff.
(0.13 MB DOC)

Table S1 Detailed information for all 1593 trans-eQTL band / FGS enrichments.
(0.93 MB XLS)

Table S2 Detailed information on all 2860 novel gene annotations inferred by Gene Atlas correlation.
(0.40 MB XLS)

Table S3 SNPs discovered in the CCNH gene across 20 inbred mouse strains.
(0.02 MB XLS)
