# Supplementary figures and images for: Gene Set Enrichment in eQTL Data Identifies Novel Annotations and Pathway Regulators
Source: PLoS Genet. 2008 May 9;4(5):e1000070. doi: 10.1371/journal.pgen.1000070 (PMC2346558; doi:10.1371/journal.pgen.1000070)

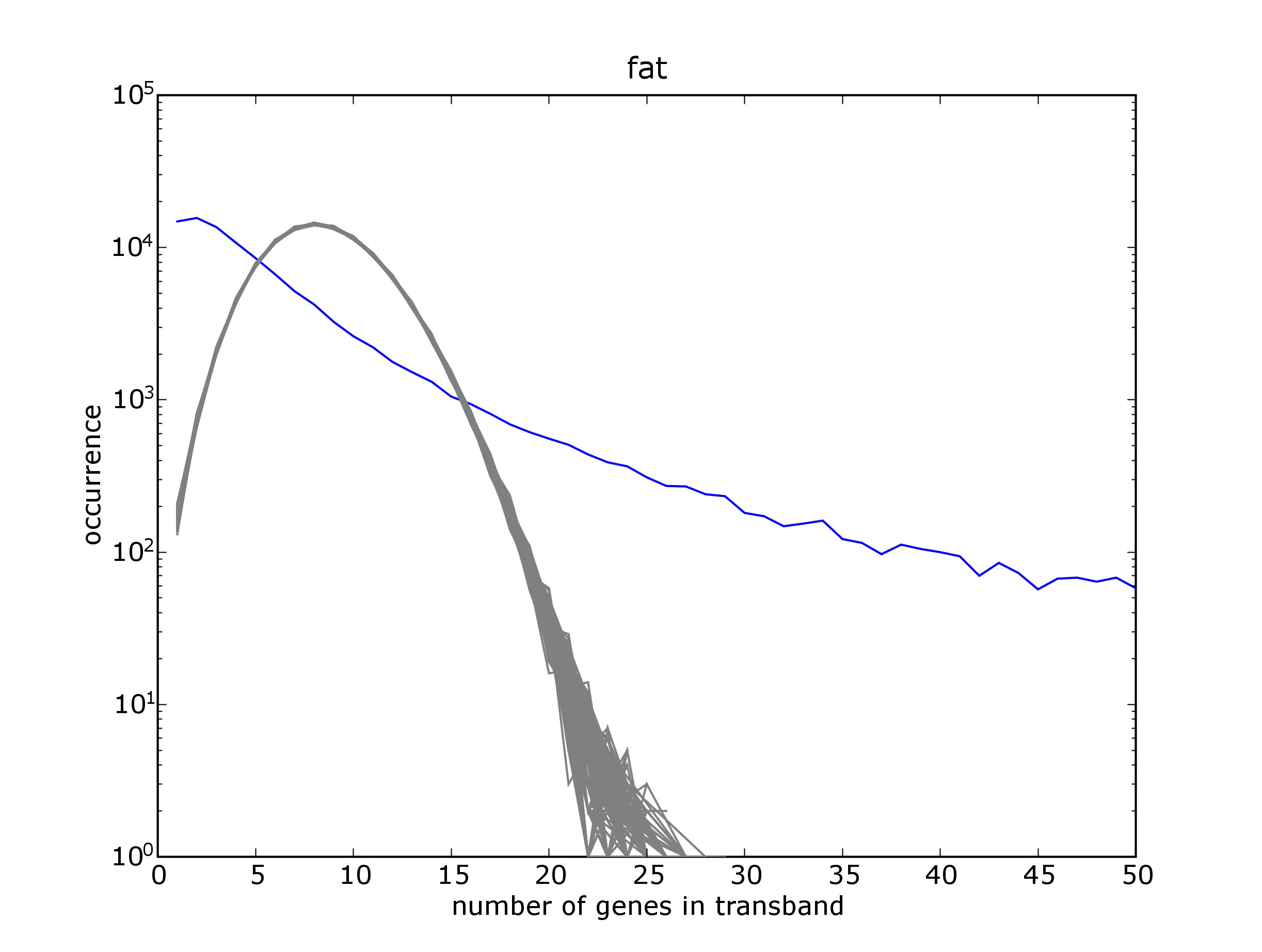

Supplement: Figure S1 — Histograms of the number of trans-band targets in a trans-eQTL band formed by chance in fat eQTL data. The number of trans-band targets in each trans-eQTL band was computed, and a histogram of trans-eQTL band sizes is shown in blue. For comparison, the QTL location of each association in the eQTL matrix was permuted 1000 times, and each gray line represents the histogram of trans-eQTL band sizes of a permuted eQTL result. (0.17 MB DOC) [file pgen.1000070.s001.doc]

**
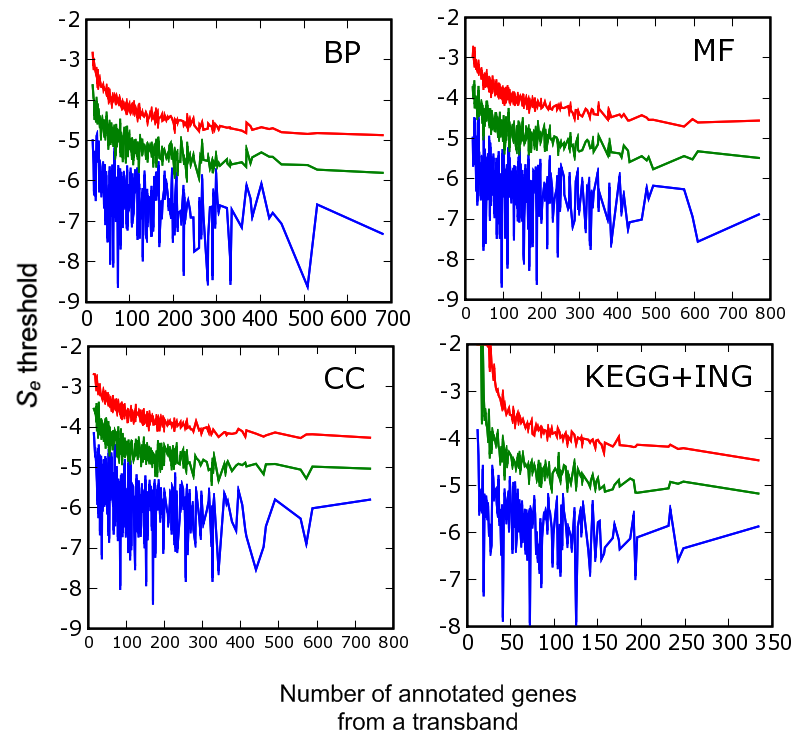
**

Supplement: Figure S2 — Enrichment score threshold for multiple testing correction over all FGS. At each size of trans-eQTL band (x-axis), 1000 random permutations of association scores was generated. For each permutation, enrichment calculation was performed for all FGS, and the maximum enrichment score was recorded. Recorded values were used to define enrichment score thresholds at 0.001, 0.01 and 0.05 (blue, green, and red, respectively) adjusted for multiple testing over all FGS. These background distributions were used to calculate adjusted enrichment p-values (adj. P). (0.16 MB DOC) [file pgen.1000070.s002.doc]

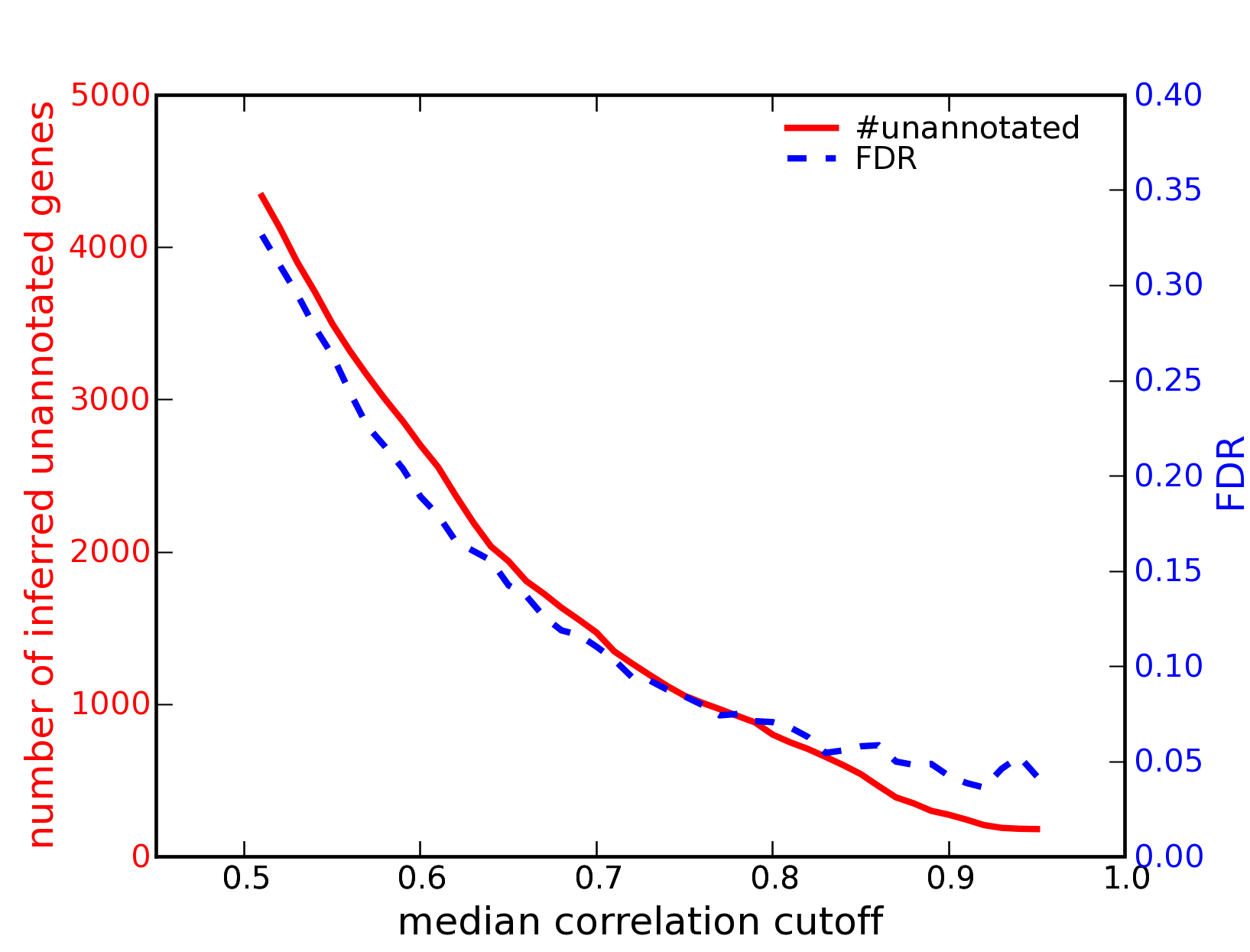

Supplement: Figure S3 — Number of inferred annotations and false discovery rate (FDR) as a function of median correlation threshold. The median correlation cutoff was used to define a threshold of inferring missing annotation. Initial settings were chosen corresponding to a 20% FDR at 0.59 cutoff. (0.13 MB DOC) [file pgen.1000070.s003.doc]
